# Predictive Factors for the Development of Gallbladder Necrosis

**DOI:** 10.7759/cureus.68310

**Published:** 2024-08-31

**Authors:** Sandeep Maharajh, Joshua Brown, Jakub Chmelo, Pooja Prasad, Alexander W Phillips

**Affiliations:** 1 Northern Oesophago-Gastric Unit, Newcastle upon Tyne Hospitals NHS Foundation Trust, Newcastle upon Tyne, GBR

**Keywords:** cholecsystitis, smoking status, c-reactive protein, white cell count, cholecystectomy, predictive factors, gallbladder necrosis

## Abstract

Introduction

Acute cholecystitis is a common complication of gallstone disease. Likewise, gallbladder necrosis is a complication of cholecystitis associated with higher risks of morbidity and mortality. Identification of risk factors which portend to gallbladder necrosis is key in prioritizing the management of higher-risk patients. This study aimed to identify such factors that predict the development of gallbladder necrosis.

Method

A retrospective review of all patients undergoing emergency cholecystectomy in a tertiary hospital over a two-year period was performed. Gallbladder necrosis was diagnosed on histopathological examination of operative specimens. Multivariable logistic regression was performed to determine risk factors for gallbladder necrosis.

Results

A total of 163 patients underwent acute cholecystectomy and 43 (26%) had proven gallbladder necrosis. Multivariable analysis demonstrated that elevated white cell count (WCC) (OR 1.122, 95%CI 1.031-1.221, p=0.007), elevated C-reactive protein (CRP) (OR 1.004, 95%CI 1.001-1.008, p=0.022) and positive smoking status (OR 5.724, 95%CI 1.323-24.754, p=0.020) were independently predictive of gallbladder necrosis. Notably, advancing age, elevated BMI, diabetes mellitus or American Society of Anesthesiologists (ASA) grade were not found to be associated with developing necrosis.

Conclusion

Patients at risk of gallbladder necrosis include those with higher WCC, CRP, and active smokers. Given the increased potential complications, these risk factors should be identified early in the management of those admitted with gallstone disease to ensure such patients receive aggressive medical therapy alongside timely and guided surgical intervention.

## Introduction

The prevalence of gallstone disease has been estimated at 15-25% of the general population [1]. In patients with cholelithiasis, 1-2% will develop cholecystitis per year, with a further estimated rate of gangrenous cholecystitis reported in 2-40% of cases of acute cholecystitis [2-4]. With approximately 66,000 cholecystectomies performed each year within the United Kingdom’s National Health Service (NHS), it has been reported as the most common major surgery performed within the discipline of General Surgery and therefore represents a significant burden of disease on the health service [5,6]. 

The clinical course of calculous cholecystitis follows obstruction of the cystic duct with subsequent inflammation of the gallbladder wall and bacterial translocation. Acalculous cholecystitis usually occurs in critically unwell patients due to functional atonia and gallbladder ischemia and is more prevalent in patients with sepsis, major burns, major surgery, diabetes mellitus, renal disease, and those on total parenteral nutrition [7,8]. In the setting of gangrenous cholecystitis, the severity of complications and mortality rate increases significantly.

Traditionally, delayed cholecystectomy at six weeks after symptom onset and resolution of infection was standard practice, with the perceived benefit of reduced bile duct injury and decreased risk of needing to convert to an open operation [9]. However, given that the severity of infection in a proportion of patients with acute cholecystitis will progress despite optimum medical management, and with proven acute surgical outcomes, patients are now recommended to have early surgery. This is reflected in the latest Cochrane review and Tokyo guidelines which advocate early laparoscopic cholecystectomy within 72 hours, or up to seven days of symptom onset [9,10]. The benefits of this approach have been demonstrated with respect to the cost of treatment, shorter hospital stays, and fewer complications [10]. Now that the standard of care is that of cholecystectomy performed at index admission, the need has arisen for the early identification of higher-risk patients such as those who have developed gallbladder necrosis. Furthermore, the provision of an emergency general surgery service for early cholecystectomy requires appropriately trained surgeons and is resource intensive. Centres admitting such patients must, therefore, be appropriately equipped to manage such cases [11].

This study assesses patients undergoing emergency cholecystectomy over a two-year period at a tertiary health institution in the United Kingdom. The aim of the study is to evaluate and determine any potential factors which may predict gallbladder necrosis and potentially help identify patients who may be less likely to respond to medical management alone.

This study was previously presented as a meeting abstract at the 2024 Surgical Research Society Annual Meeting on January 12, 2024. 

## Materials and methods

A retrospective observational study was conducted over the period May 2021 to May 2023 utilizing the hospital’s electronic health records. All patients presenting to a tertiary referral hospital, Royal Victoria Infirmary, Newcastle Upon Tyne, United Kingdom, who were acute surgical admissions and subsequently underwent emergency cholecystectomy on their index admission were included. There were no exclusion criteria. Each patient was investigated preoperatively with baseline blood tests including full blood count, C-reactive protein (CRP), and liver function tests. Radiological investigations were performed with either abdominal ultrasound or computer tomography (CT) scanning to confirm diagnosis of gallbladder disease. Magnetic resonance imaging (MRI) scan was done if clinically indicated, for example, if there was an indication of biliary tree abnormalities or obstructive cholestasis on liver function blood tests. The decision for surgical intervention with cholecystectomy was based on the discretion of a biliary-competent surgeon. The main indications were of those patients presenting with suspected biliary colic with refractory symptoms, as well as those with raised inflammatory markers and/or clinical signs of acute cholecystitis. All surgeries were attempted laparoscopically. Operation notes were reviewed for each patient to document intraoperative findings and histopathological reports of intraoperative specimens taken were reviewed to determine which cases had confirmed gallbladder necrosis.

Specific parameters analyzed included patient factors such as age, sex, comorbidities, body mass index, American Society of Anesthesiologists (ASA) status, and tobacco use. Patients were considered immunocompromised if there was current use of immunomodulating medications or chemotherapy for autoimmune conditions or malignancy. Patients with type 2 diabetes mellitus, diet control and insulin dependence, and type 1 diabetes mellitus were included. Preoperative factors included white cell count (WCC), CRP and the presence of gallstones.

Statistical analysis was performed with IBM SPSS Statistics for Windows, Version 29.0 (Released 2023; IBM Corp., Armonk, New York, United States) to determine the statistical significance of the above variables for independently predicting the outcome of gallbladder necrosis. Categorical variables were compared with Chi-square test or Fisher's test as appropriate. Continuous variables were compared with the Mann-Whitney U test. Multivariable logistic regression was performed with variables with p-values lower than 0.1 in the univariable testing. Receiver operation curve (ROC) analysis was conducted for continuous variables with p-values less than 0.05 in the multivariable model. A p-value lower than 0.05 was deemed statistically significant throughout.

## Results

Over the two-year period, a total of 163 patients presented with symptoms of gallbladder disease and subsequently underwent emergency cholecystectomy at our hospital. Female patients constituted most of the cohort at 102 (63%). As shown in Table 1, the median age (interquartile range (IQR)) for necrotic and non-necrotic gallbladders were 59 years (47-69) and 53 years (36-63), respectively. Of the patients, 152 (93%) had radiologically confirmed gallstones prior to surgery based on either CT or ultrasound imaging and corroborated with histopathological examination. Clinical indication for surgery included cholecystitis in 145 (89%) patients with the remainder having biliary colic not managed with regular opioid analgesia. The median number of days from admission to surgery was one day (range 0-5 days). Of the 18 patients who underwent surgery for biliary colic, 17 (94%) had either acute or chronic cholecystitis confirmed on histopathological examination; two of these 17 cases also reported gallbladder necrosis.

**Table 1:** Clinicopathological characteristics of patients with and without gallbladder necrosis. Data given as number (percentage) and wherever marked, as median (IQR)). Statistically significant p-value given in bold. BMI, body mass index; ASA, American Society of Anesthesiologists physical status classification; WCC, white cell count; CRP, C-reactive protein; IQR, interquartile range

Variables		No necrosis	Necrosis	p-value
Age (years), median (IQR)		53 (36-63)	59 (47-69)	0.035
Sex, male		39 (32.8)	21 (48.8)	0.062
BMI (kg.m^-2^), median (IQR)		30.95 (27.47-36.25)	32.95 (27.42-38.09)	0.229
Smoker, yes		4 (3.3)	6 (14.0)	0.013
Diabetic, yes		8 (6.7)	7 (16.3)	0.061
Immunocompromised, yes		2 (1.7)	2 (4.7)	0.574
ASA	1	26 (21.7)	4 (9.3)	0.192
	2	72 (60.0)	29 (67.4)	
	3	22 (18.3)	10 (23.3)	
WCC, median (IQR)		11.32 (7.49-15.03)	16.84 (12.49-21.43)	<0.001
CRP, median (IQR)		37 (8.5-105.8)	195 (69-280)	<0.001
Calculous, yes		111 (92.5)	40 (93.0)	0.910
Time admission to operation (days)		1 (1-2)	1 (1-2)	0.274

The surgical approach for cholecystectomy was laparoscopic in all cases but conversion to open surgery occurred in six (4%) of the cases; four patients had a conversion to open surgery due to challenging anatomy with chronic inflammatory adhesions and two patients had intraoperative bleeding which could not be controlled laparoscopically. Macroscopically, 27 (17%) were identified as necrotic based on intraoperative findings while histopathological reports proved 43 (26%) of the intraoperative specimens to contain necrotic gallbladder tissue.

Patients with confirmed gallbladder necrosis were older (median age of 59 vs 53 years, p=0.035) and were more likely smokers (14.0% vs 3.3%, p=0.013). Their median (IQR) WCC at presentation was higher than in those without gallbladder necrosis (16.84 x 10^9^/L (12.49-21.43) vs 11.32 x 10^9^/L (7.49-15.03), p<0.001). Similarly, this was observed for CRP where the median (IQR) in the gallbladder necrosis group was 195 mg/L (69-280) vs 37 mg/L (8.5-105.8) in those without gallbladder necrosis (p<0.001). Other studied factors were similar in both groups (Table 1).

Multivariable analysis demonstrated that smoking status (OR 5.724, 95%CI 1.323-24.754, p=0.020), raised WCC (OR 1.122, 95%CI 1.031-1.221, p=0.007) and elevated CRP (OR 1.004, 95%CI 1.001-1.008, p=0.022) were independently predictive of gallbladder necrosis (Table 2).

**Table 2:** Multivariable logistic regression for variables with p< 0.1 in univariable testing. OR, odds ratio; CI, confidence interval; WCC, white cell count; CRP, C-reactive protein

Variables	OR	95% CI	p-value
Age	1.005	0.977-1.034	0.733
Sex, male	1.176	0.480-2.880	0.723
Smoker, yes	5.724	1.323-24.754	0.020
Diabetic, yes	1.727	0.458-6.507	0.419
WCC	1.122	1.031-1.221	0.007
CRP	1.004	1.001-1.008	0.022

ROC analysis revealed an area under the curve (AUC) for WCC of 0.768 (95%CI 0.691-0.844) and for CRP of 0.773 (95%CI 0.691-0.855). 

## Discussion

The preoperative diagnosis of gangrenous cholecystitis remains a challenge and takes account of a patient’s medical history, physical examination, laboratory investigations, and radiological scan results. However, aside from postoperative histopathological confirmation, a definitive preoperative diagnostic criterion remains elusive. This retrospective observational study assessed patient-specific and preoperative factors to identify potential predictors which increase the likelihood of gallbladder necrosis being present. This study aims to contribute to existing published literature on gallbladder necrosis by presenting our experience in emergency cholecystectomies at a tertiary health care institution, analysing preoperative factors in our population and determining potentially independently predictive factors for necrosis. It was found that elevated WCC, elevated CRP, and positive smoking status were independent predictors of gallbladder necrosis.

Over the two-year study period, there were 163 emergency cholecystectomies performed. The rate of conversion from laparoscopic surgery to open procedure was 4%, with necrotic gallbladders being found in half of the converted cases. Historically, conversion rates for cholecystectomy have been reported as high as 15%; however, recent national data of combined elective and emergency cholecystectomy cases in the United States by Shah et al. reported conversion rates of 1.89% [12] and single subspecialist reviews reporting conversion rates as low as 0.49% [13,14]. In cases of gangrenous cholecystitis, the conversation rate from laparoscopic to open cholecystectomy has been reported to be as high as 35% [15]. Uncontrolled bleeding, distorted anatomy due to chronic inflammation with fibrosis, and dense adhesions to surrounding structures are potential challenges in laparoscopic surgery which can prompt conversion to open surgery [13].

Other similar studies have cited atherosclerosis as a predisposition to gallbladder necrosis and have found risk factors such as diabetes mellitus and cerebrovascular disease as predictive [4,16]. In this cohort of patients, a positive smoking status was found to be an independent risk factor for gallbladder necrosis. Other patient factors such as age, elevated BMI, and ASA grade have not been found to be predictive of the diagnosis. In the largest retrospective review on the topic to date of 5,812 patients, Wu et al. similarly found ASA grade as not statistically significant but concluded that advancing age was a predictor of necrosis on multivariable analysis [17], in keeping with several other studies [4,18-22].

Specific preoperative blood markers have been found to be independent predictors of gallbladder necrosis in this study. Analysis of elevated WCC with ROC analysis produced an AUC of 0.768. In keeping with the findings of the above-cited literature, elevated WCC was found to be predictive at varying thresholds between 13 and 15 x 10^9^/L [17,21]. Another inflammatory marker, elevated CRP was found to be predictive with an associated AUC value of 0.773. Similarly, Mok et al. found CRP to have a high predicting value in their retrospective study of 141 patients but found that WCC was not predictive [23]. Nikfarjam et al., in a review of 290 patients, found elevated CRP and WCC to be associated with necrosis on univariable analysis [21]. A study of the literature by Raffee et al., however, did not conclude that WCC or CRP was predictive of gallbladder necrosis [24]. Larger, prospective studies are needed for firmer conclusions with respect to the predictive power of pre-operative inflammatory markers. The presence of gallstones or lack thereof (calculous vs acalculous cholecystitis) had no significance in predicting whether necrosis was present at the time of operation in this study.

The incidence of gallstones varies geographically and by ethnicity with an approximately 15% incidence in European populations and up to 70% in the Native American population [25,26]. While the mortality risk for those with gallstones has been estimated at 0.6%, given its rising incidence, a significant potential for complications and mortality exists within the context of a national healthcare system [26]. Of the 1-2% of patients who develop symptomatic cholelithiasis each year, patients most commonly present with abdominal pain, or less commonly with signs of infection to suggest acute cholecystitis [25]. This can progress to gangrenous cholecystitis, empyema, and perforation [4]. Gangrenous cholecystitis has been reported to be associated with greater risks of complications such as abscess formation, perforation, biliary peritonitis, increased rates of conversion from laparoscopic to open surgery, increased ICU admissions, longer hospital stays and death [18,19,27,28]. It is clear that increased clinical suspicion of gangrenous cholecystitis is necessary to ensure that such patients are appropriately diagnosed and surgical intervention is prioritized in order to reduce rates of morbidity and mortality.

The significance of this study also extends to critically unwell patients with suspected gangrenous cholecystitis who may be unfit for laparoscopic surgery and general anaesthesia. Percutaneous cholecystostomy (PC) is an alternative to cholecystectomy. Although not curative for gallbladder disease, it allows for sepsis control and symptom management. Terrone et al. proved that PC had no difference in morbidity or mortality compared to cholecystectomy in severe acute cholecystitis, but had higher rates of readmission [29]. Therefore, in stratifying patients at higher risk of gangrenous cholecystitis, the safest case-specific intervention should be considered in a timely manner. 

It should be highlighted that preoperative WCC and CRP are continuous variables. Through this study, it has been determined that elevations of these variables should raise the suspicion of gallbladder necrosis being present. In the statistical analysis of the data, ROC analysis was utilized to determine the diagnostic performance of these variables and produced an AUC of 0.768 for WCC and 0.773 for CRP.

Some study limitations need to be noted. Firstly, this review utilized a modest patient sample size, and larger numbers would allow greater power of the predicting variables cited. Secondly, this study included patients being managed in a single centre. Therefore, the heterogeneity of patient demographics may be decreased in terms of socioeconomic status, health-related behaviours, ethnicity, and educational background. Thirdly, pathological examination of intraoperative specimens entailed sectioning and fixation, which can result in slides which are not fully representative of the entire pathology of the gallbladder and thus, rates of necrosis could be under-reported. Furthermore, the results of our ROC analysis do not identify a specific threshold for WCC and CRP since a limitation is looking at laboratory investigations preoperatively instead of analyzing blood infection markers at a fixed point in time since symptom onset. Patients present at varying stages of diseases of the gallbladder, at varying severity of symptoms, and therefore surgery may occur before infection markers peak. Overall, however, it is a useful predictive factor, in addition to smoking status, for necrosis.

During the study period, not all patients presenting with symptomatic gallbladder disease were operated on and the decision to proceed with surgery varied given that the study site is a multi-surgeon centre. Our centre does not operate a dedicated emergency gallbladder operating list and surgeons include both hepato-pancreato-biliary specialists and other specialist general surgeons. This has several effects on patient management including challenges in accessing timely emergency theatre services and varying thresholds in the decision-making process to proceed with surgery. This would therefore have an unknown result on the studied variables and statistical analysis results if this additional set of patients were operated on and included. In addition to the improved clinical outcomes of index admission emergency cholecystectomy compared to delayed cholecystectomy, there are established financial savings for hospitals and improved quality-adjusted life years for patients [30]. Despite these benefits, the lack of dedicated theatre lists is still a reality faced by some hospitals in managing symptomatic gallbladder disease.

Despite these limitations, the findings of this study can be extended to the wider population in a mature health system. The study focuses on a common presentation, along with an established management framework to allow health professionals to draw comparisons in their own practice. Furthermore, the definitive diagnosis of gallbladder necrosis was recorded based on pathology reports, including specimens from patchy necrosis to transmural necrosis. The avoidance of this criteria based on intra-operative diagnosis removes possible inter-surgeon variability in the clinical diagnosis of gangrenous cholecystitis.

## Conclusions

Positive smoking status, elevated WCC, and elevated CRP have been found to be independently predictive of gallbladder necrosis. Gallbladder necrosis is a complication of gallbladder disease associated with higher risks of morbidity and mortality. Increased awareness of potential risk factors to aid in the clinical diagnosis of such patients can allow prioritization of aggressive medical therapy and timely surgical intervention.
